# Will the fibers of the future be considered fibers?

**DOI:** 10.1093/nsr/nwae235

**Published:** 2024-07-08

**Authors:** Dewen Xu, Ya Liu, Hailiang Wang, Wei Yan, Changjun Jiang, Meifang Zhu

**Affiliations:** State Key Laboratory for Modification of Chemical Fibers and Polymer Materials, College of Materials Science and Engineering, Donghua University, China; State Key Laboratory for Modification of Chemical Fibers and Polymer Materials, College of Materials Science and Engineering, Donghua University, China; State Key Laboratory for Modification of Chemical Fibers and Polymer Materials, College of Materials Science and Engineering, Donghua University, China; State Key Laboratory for Modification of Chemical Fibers and Polymer Materials, College of Materials Science and Engineering, Donghua University, China; Key Laboratory of Embedded System and Service Computing, Ministry of Education, Tongji University, China; Department of Computer Science and Technology, Tongji University, China; State Key Laboratory for Modification of Chemical Fibers and Polymer Materials, College of Materials Science and Engineering, Donghua University, China

## Abstract

This work explores the pivotal breakthroughs and historical developments in fibers over the past century, while also identifying future research directions and emerging trends that promise to shape the future of this field.

Fiber materials are key materials that have changed human history and promoted the progress of human civilization. In ancient times, humans used feathers and animal skins for clothing, and later they widely employed natural fibers such as cotton, hemp, silk and wool to make fabrics (Fig. 1a). Chinese ancestors had mastered the art of natural fiber weaving as early as the Neolithic Age. Two thousand years ago, people were already familiar with and adept at techniques for spinning natural fibers [1]. Around 200 BCE, China opened up the Silk Road, facilitating the transportation of high-quality silk, which had a profound impact on global civilization and culture. With the advancement of science and technology, synthetic and artificial fibers emerged, leading to rapid development in the chemical fiber industry, profoundly altering people's lives. Today, the field of fiber science is undergoing revolutionary progress and development. Fibers, characterized by significant innovations in components, structures, properties, and functionalities, are transcending conventional roles in clothing and aesthetics. In this perspective, we dissect the classic breakthroughs and development of fiber materials over the past century, and pinpoint the future research directions and development trends of advanced fiber materials that will further shape the future of the world.

**Figure 1.**
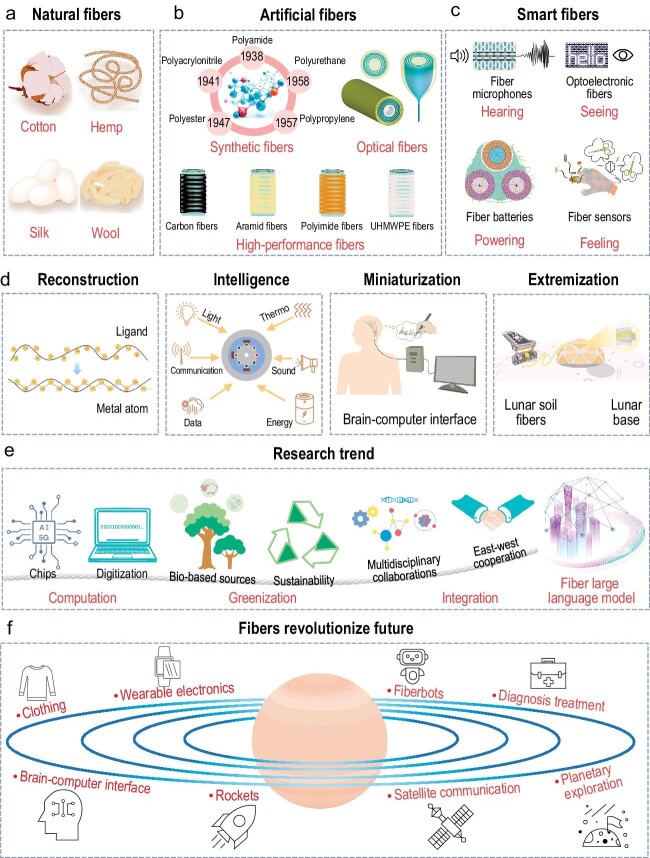
(a) Natural fibers. (b) Artificial fibers. (c) Smart fibers with the capabilities of hearing, seeing, powering, and feeling. (d) The current development of fiber exhibits distinctive characteristics: reconstruction, intelligence, miniaturization, and extremization. (e) The three research directions of fibers including computation, greenization, and integration. (f) Fibers have the ability to revolutionize the future.

In the 1930s, American chemist Dr. Wallace Hume Carothers, while working at DuPont, pioneered the development of nylon, the world's first synthetic fiber. This breakthrough heralded the dawn of the synthetic fiber era. Subsequently, polyacrylonitrile, polyester, polypropylene, and polyurethane were developed in both the United States and Europe. These comprise the five major synthetic fibers (Fig. 1b), profoundly transforming industries spanning from fashion to engineering. In 1966, Chinese-born scientist Charles Kao proposed and confirmed the transmission of photons through glass fibers (optical fibers) [2]. Optical fiber serves as the cornerstone of the modern information age, underpinning essential technologies such as the Internet, the Internet of Things (IoT), big data, cloud computing, machine learning, and artificial intelligence (AI) (Fig. 1b). Kao was awarded the Nobel Prize in Physics in 2009 and is regarded as the father of optical fiber communication. Meanwhile, in order to meet the strategic security needs of national defense, military, and aerospace, high-performance fibers such as carbon fibers, aramid fibers, ultra-high-molecular-weight polyethylene fibers, and polyimide fiber have achieved breakthrough development (Fig. 1b).

Developed countries like the United States, Japan, and Germany have recognized the revolutionary impact that the next generation of fibers will bring (Fig. 1c) [3–5]. Various initiatives have been established to advance fiber development. One example is Advanced Functional Fabrics of America (AFFOA) that was established in the United States in 2015 to focus on revolutionizing the fiber and textile industry by leveraging cutting-edge technology and innovation. In 2019, Prof. Meifang Zhu at Donghua University, conceived the concept of FIBER (Functional, Integrated, Brainy, Electronic, Responsive) which pinpoints the key necessary attributes of smart fibers [1]. The current development of advanced fiber materials exhibits distinctive characteristics: reconstruction, intelligence, miniaturization, and extremization (Fig. 1d). Overturning the structure of traditional fibers’, reconstructed fibers incorporate controlled structures of organic monomers, inorganic and metallic units that achieve totally new structures, properties, and functions. For example, reconstructed metal-backboned polymers (MBPs) have been proposed [6]. Fibers’ intelligence implies a paradigm shift in the capabilities of fibers. These fibers can actively interact with their environment and users, and can sense, process, and transmit data, opening up a world of possibilities across various fields. Prof. Yoel Fink at MIT has proposed multimaterial fibers that integrate metals, semiconductors, functional polymers, and even microelectronic devices [7]. Fibers that see, hear, sense, communicate, store, and harvest energy, have been subsequently realized. Miniaturization of fibers refers to the process of reducing the size of fibers to smaller dimensions, often to the micro- or nanoscale, targeting innovative applications in aerospace, optics, healthcare, and beyond, where size and weight do matter a lot. For example, miniaturized fiber-shaped surgical instruments, such as flexible endoscopes, catheters, and fiberbots, possess intuitive features for minimally invasive surgery, thus catalyzing the rapid development of precision medicine [8]. Innovatively designed and manufactured on a large scale, miniaturized metal fibers, as small as 40 nm, provide a cutting-edge platform for brain–computer interfaces [9]. Fiber's extremization refers to the capability of fibers to function and perform under extreme conditions such as high/low temperature fluctuation, vacuum, and radiation in space. Advanced fiber materials are playing a crucial role within material systems across space research, space manufacturing, and space applications. For example, space habitats and satellites are now equipped with unprecedented acoustic fibers designed to monitor impacts from high-velocity space particles and debris [10].

Rapid advancements in fiber materials have led to the emergence of new technologies and applications. To further promote the development of advanced fiber materials, we propose three research directions including computation, greenization, and integration (Fig. 1e). First, when we continuously incorporate circuits, chips, and central processors into fibers, seamlessly integrating them with 5G and AI, we can create what are known as computing fibers. With the creation of the ‘fiber large model’, such fibers not only sense and act, but also are digitalized and capable of computing. Second, since most current fibers are made from synthetic materials, transitioning to fiber materials derived from bio-based sources will facilitate a green transformation in both the fiber industry and academia. This transition aims to achieve the recyclability, sustainability, and substitutability of fibers, thus contributing to the attainment of the ‘dual carbon’ or carbon neutrality goal. Third, the concept of ‘integration’ underscores the necessity for multidisciplinary collaborations, encompassing fields from physics, chemistry, and materials science to mechanical engineering, electrical engineering, biomedical engineering, and computer science. Moreover, in light of the current international situation, it is crucial to adopt an open-minded approach, foster extensive academic cooperation between East and West, establish collaboration platforms, and drive transformative development in both the fiber industry and academia.

Fibers have played transformative roles in every historical period, and they still keep changing. In the context of planetary exploration, fibers are crucial construction materials in developing rockets, interstellar bases, and ensuring stable satellite communication. In Smart Living, wearable electronic devices are revolutionizing how we manage our lives, where fibers are serving as their fundamental unit. Delving deeper into the microscopic realm, fiberbots and fiber-constructed brain–computer interfaces are employed to understand and control complex systems within the human body. From the macro universe to the micro world, today's fibers have evolved far beyond their historical counterparts. The question remains: will the fibers of the future still be considered fibers (Fig. 1f)?
